# Data of pre-and post-operative images and video of marsupialization of congenital vallecular cyst by coblation technique

**DOI:** 10.1016/j.dib.2020.105689

**Published:** 2020-05-19

**Authors:** Eric Ma, Krishna Revanna Gopagondanahalli, Jenica Su-ern Yong, Chua Mei Chien, Suresh Chandran

**Affiliations:** a KK Women's and Children's Hospital, Singapore; b NUS Yong Loo Lin School of Medicine, Singapore; c Duke-NUS Medical School, Singapore; d Lee Kong Chian Imperial School of Medicine, Singapore

**Keywords:** Vallecular cyst, Stridor, Coblation, Marsupialization, Laryngoscopy, Congenital, Newborn

## Abstract

This data describes the modern surgical treatment of congenital vallecular cyst in a term newborn infant who developed neonatal stridor on day 1 of life. Diagnosis was made by nasoendoscopy and the infant underwent successful treatment by marsupialization via coblation technique. Images and videos were taken during the procedure both pre and post-operatively. This case highlights the need for an interdisciplinary evaluation of persistent neonatal stridor in newborn infants for early diagnosis and intervention to avoid critical airway obstruction and potentially fatal outcomes, DOI: 10.1016/j.epsc.2020.101460[1].

Specifications table

**Table:** 

Subject	Otorhinolaryngology and Facial Plastic Surgery
Specific subject area	Congenital anomaly of the airway presenting with progressively increasing stridor in a newborn infant on day1 of life
Type of data	Images and Video
How data were acquired	Microlaryngoscopy and bronchoscopy
Data format	Raw
Parameters for data collection	Pre- and post-operative sessions: Images were obtained in JPEG format and processed to 300 dpi. Videos were recorded in MP4 format at 25 frames per second.
Description of data collection	Images and videos were taken during laryngoscopy and marsupialization of the vallecular cyst by coblation.
Data source location	Institution: KK Women's and Children's Hospital City: Singapore Country: Singapore 229899
Data accessibility	Data accessible:
	- With the article published in Journal of Pediatric Surgery Case Reports, DOI: 10.1016/j.epsc.2020.101460 [1]. - More images and videos are available in the Data In Brief journal.
Related research article	Ma E, Gopagondanahalli KR, Yong JS, Chua MC, Chandran S. Congenital Vallecular Cyst causing severe inspiratory stridor in a newborn. Journal of Pediatric Surgery Case Reports. DOI: 10.1016/j.epsc.2020.101460

## Value of the Data

• Congenital vallecular cyst is a rare cause of neonatal stridor, which could be fatal unless appropriate evaluation and intervention are done early.
• Increasing awareness of this rare congenital anomaly among the neonatal fraternity can facilitate early involvement of otolaryngologist, allowing quick assessment and required intervention.
• Pre- and post-intervention images and video of the vallecular cyst give a clear illustration of this congenital anomaly and the newer modality of surgical treatment for trainees in otolaryngology and pediatrics.

## 1 Data Description

**Fig. 1:**
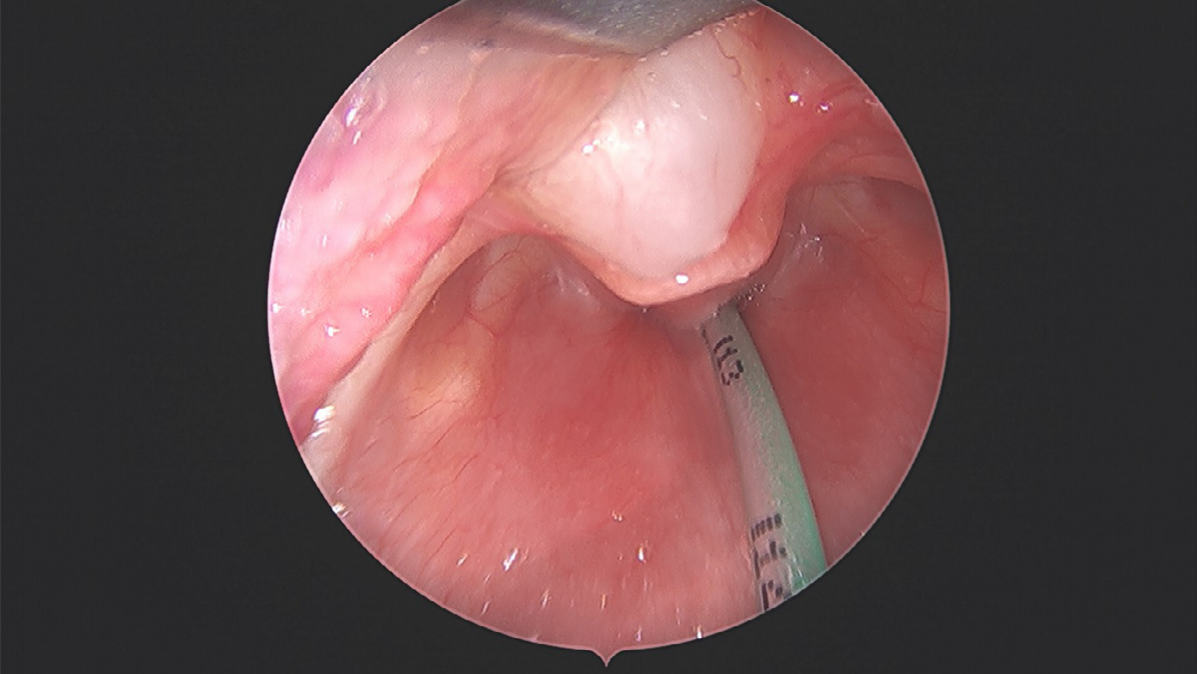
Endoscopic view of vallecular cyst with oro-gastric tube in-situ.

**Fig. 2:**
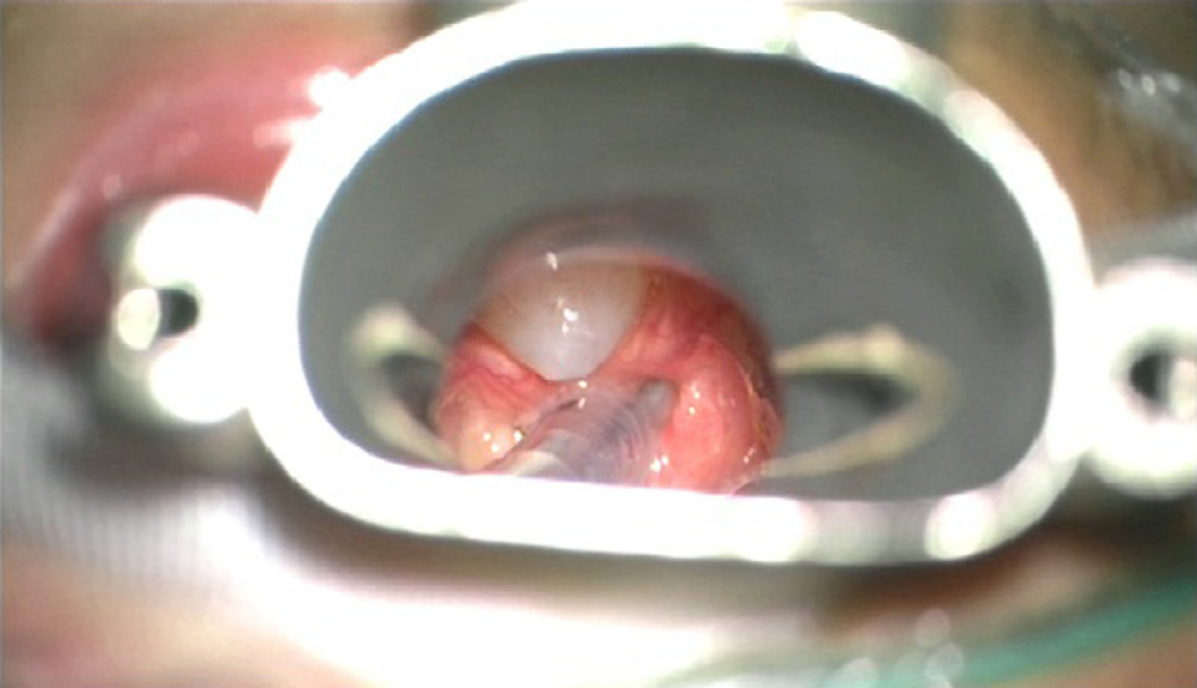
Pre-operative view of vallecular cyst at 11o'clock position seen through the Lindholm laryngoscope.

**Fig. 3:**
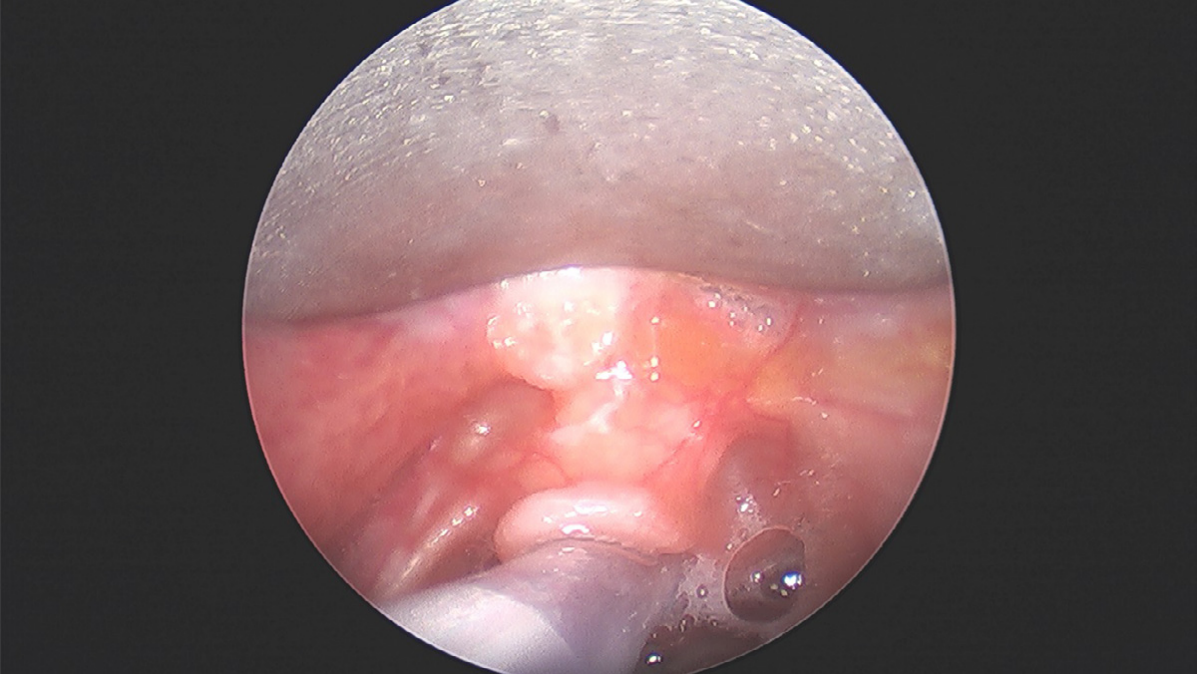
Intra-operative view of vallecular cyst after marsupialization. Endotracheal tube in-situ.

The data was collected from a term female newborn infant who presented with neonatal stridor. The infant presented with respiratory distress at 12 hours of life and required admission to neonatal intensive care unit for continuous positive airway pressure support. On day 2 of life, the respiratory distress persisted but the chest x-ray did not reveal any significant lung disease. Hence, an upper airway evaluation was undertaken by the otolaryngologists. Flexible nasoendoscopy showed a cystic mass at the vallecula abutting the epiglottis (Fig. 1). An urgent microlaryngoscopy and bronchoscopy was performed to confirm the diagnosis. Thereafter marsupialization of the vallecular cyst was performed using coblation technique (Video). Images of the vallecular cyst before and after marsupialization were taken during the procedure (Fig. 2, 3). The infant was extubated soon after the procedure and was stable in continuous positive airway support. She was fed within 48 hours post-operatively and was depronged to room air. She was discharged home well on day 5 of life. Additional videos of the procedure may also be viewed in the Journal of Pediatric Surgery Case Reports [1].

## 2 Experimental Design, Materials, and Methods

A Parsons laryngoscope size 8 (Karl Storz, Tuttlingen, Germany) was used together with a Storz xenon light source for the laryngoscopy. A rigid 4mm telescope was mounted with Stryker endoscopic camera (Stryker, Kalamazoo, Michigan, US). Images and videos were captured with Stryker video tower. After confirmation of the diagnosis, a Lindholm laryngoscope was suspended and microscope was used during surgery. Raw images and videos were obtained.

## Appendix Supplementary materials

Video Intraoperative endoscopic video of removal of vallecular cyst via coblation. Image, video 1
